# Unveiling the Chemical Profile of *Teucrium chamaedrys* subsp. *gracile* (Batt.) Rech.F. dried Aerial Parts From Algeria

**DOI:** 10.1002/cbdv.202500236

**Published:** 2025-07-17

**Authors:** Claudio Frezza, Daniela De Vita, Chiara Dal Bosco, Khellaf Rebbas, Hamdi Bendif, Stefania Garzoli

**Affiliations:** ^1^ Dipartimento Di Scienze della Vita, Della Salute e delle Professioni Sanitarie Università Degli Studi Link Campus Rome Italy; ^2^ Dipartimento Di Biologia Ambientale: Università di Roma “La Sapienza” Rome Italy; ^3^ Department of Chemistry Sapienza University Rome Italy; ^4^ Department of Natural and Life Sciences, Faculty of Sciences University of M'Sila, University Pole, Road Bordj Bou Arreiridj M'Sila Algeria; ^5^ Biology Department, College of Science Imam Mohammad Ibn Saud Islamic University (IMSIU) Riyadh Saudi Arabia; ^6^ Department of Chemistry and Technologies of Drug Sapienza University Rome Italy

**Keywords:** GC‐MS, HS‐SPME, NMR, phytochemical analysis, secondary metabolites

## Abstract

In this paper, the first phytochemical analysis on *Teucrium chamaedrys* subsp. *gracil*e was reported. The dried aerial parts collected in Algeria were studied, and the analysis was conducted using different techniques: gas chromatography/mass spectrometry (GC/MS) for the fatty acid content, headspace solid‐phase microextraction‐GC‐MS for the volatile composition, column chromatography along with nuclear magnetic resonance spectroscopy, and MS for non‐volatile pattern characterization. Saturated fatty acids were found to be prevalent, with palmitic acid as the major compound. In the volatile fraction, β‐caryophyllene and humulene, comprised in the class of sesquiterpenes, were the major components. Indeed, five non‐volatile compounds were observed, namely teucrioside (**1**), cirsiliol (**2**), harpagide (**3**), and chlorogenic acid (**4**). The employed analytical techniques have proven to be suitable for producing reliable and reproducible results in the description of the phytochemical profile of the matrix.

## Graphical abstract

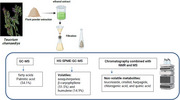

## 1 Introduction

The complete phytochemical characterization of medicinal plants has now become a fundamental aspect, reporting important information that may justify their ethnobotanical uses and biological effects, as well as providing sources of new natural compounds for the pharmaceutical, cosmetic, and nutraceutical industries.

*Teucrium* L. species, comprised in the Lamiaceae family, represent some of the most relevant medicinal plants of the world, mainly employed against digestive disorders, abscesses, gout, and conjunctivitis, and endowed with many biological effects, including antioxidant, cytotoxic, anti‐inflammatory, and antimicrobial [1]. They count almost 400 and are distributed in several regions, especially in the Mediterranean basin [1], with Algeria hosting 20 [2]. In the last years, they have undergone many botanical re‐classifications and ethnobotanical revisions following new molecular and medical discoveries that have modified the entire genus [3, 4]. In particular, for what concerns the ethnobotanical aspect, several health claims have been issued due to the presence of the strong hepatotoxic compounds known as *neo*‐clerodane diterpenoids [5, 6], which are extremely common in this genus [1].

**FIGURE 1:**
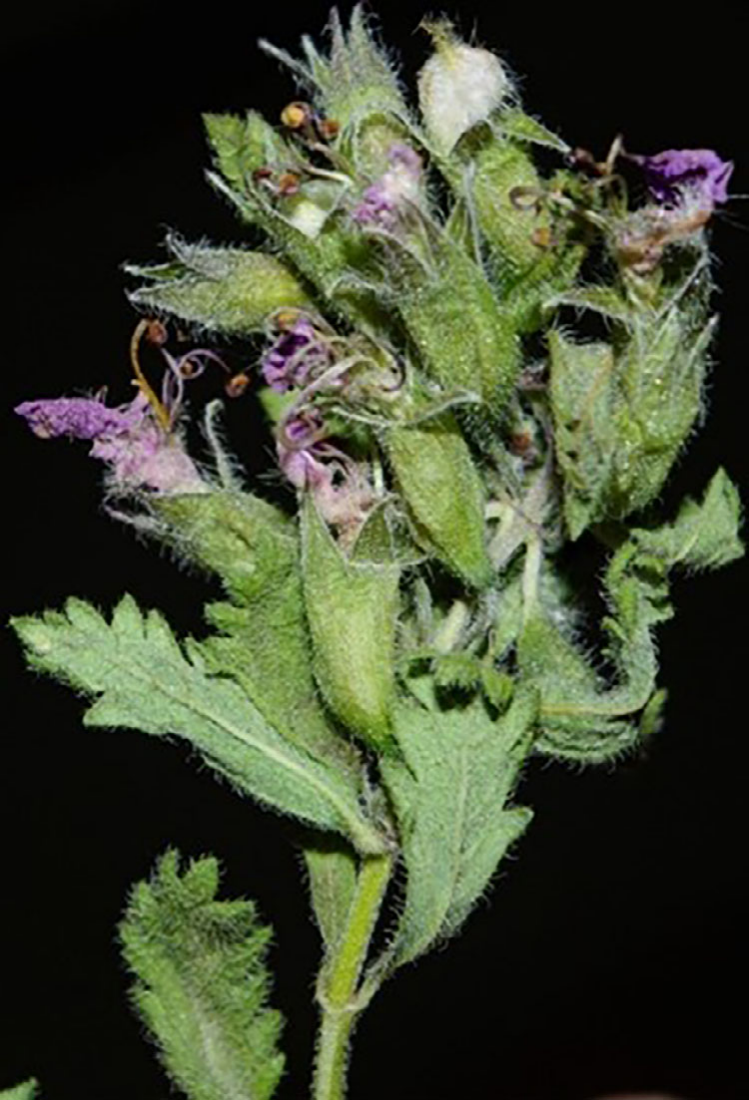
Image of *Teucrium chamaedrys* subsp. *gracile* (Batt.) Rech.f. dried aerial parts.

*Teucrium chamaedrys* subsp. *gracile* (Batt.) Rech.f., synonym of *Teucrium chamaedrys* var. *gracile* Batt. and *Teucrium chamaedrys* subsp. *maroccanum* Rech.f., (Figure 1) is a perennial herbaceous sub‐shrub endemic to Algeria and Morocco [7]. It is morphologically characterized by a hairy and branchy stem which is also woody at the base and oval leaves with softly knobbed brims and tiny blooms on branch tops [1]. With respect to the nominal species *Teucrium chamaedrys* L., the stem of *T. chamaedrys* subsp. *gracile* is thinner, from which the name [7]. This subspecies has never been studied for its phytochemical content before, but several classes of natural compounds have been already reported in the nominal species such as essential oils components [8], triterpenes [9], diterpenoids [10], phenyl‐ethanoid glycosides [10, 11, 12, 13], flavonoids [11, 14], iridoids [11, 13] and simple phenolics [11, 14]. The same classes of natural compounds have also been evidenced in other *Teucrium* species [1].

In this work, the phytochemical analysis of the dried aerial parts of *T. chamaedrys* subsp. *gracile* collected in Algeria was reported to study the fatty acid content, the volatile composition, and the non‐volatile compounds. This work provides the first comprehensive phytochemical analysis of the dried aerial parts of *Teucrium chamaedrys* subsp. *gracile* from Algeria, filling a gap in the existing literature. Previous studies focused on other subspecies or regions, making this research novel and essential for understanding the plant's chemical diversity.

## 2 Results and Discussion

### 2.1 Fatty Acid Content

The GC‐MS analysis of the dried aerial parts of *T. chamaedrys* subsp. *gracile* collected in Algeria allowed the detection of nine fatty acids (FAs) listed in Table 1.

**TABLE 1:** Fatty acid content (percentage mean value ± standard deviation) in the dried aerial parts of *Teucrium chamaedrys* subsp. *gracile* as determined by gas chromatography/mass spectrometry (GC/MS).

N°	Component ^a^	RT ^b^	LRI ^c^	LRI ^d^	(%)
1	Capric acid, C10:0 (SFA)	6.87	1370	1373	1.0 ± 0.05
2	Myristic acid, C14:0 (SFA)	9.20	1762	1759	2.1 ± 0.08
3	Palmitic acid, C16:0 (SFA)	11.98	1971	1973	54.1 ± 1.15
5	Elaidic acid, C18:1*n9* (UFA)	14.39	2143	2141	12.6 ± 0.20
6	Oleic acid, C18:1*n*9 (UFA)	14.50	2168	2171	21.1 ± 0.31
7	Stearic acid, C18:0 (SFA)	14.87	2192	2188	4.8 ± 0.08
8	Arachidonic acid, C20:4*n*6 (UFA)	17.71	2326	2324	2.3 ± 0.04
9	Behenic acid, C22:0 (SFA)	20.59	2570	2567	2.0 ± 0.02
	SUM				100.0
	Saturated FAs (SFA)				64.0
	Unsaturated FAs (UFA)				36.0

^a^ The components are reported according to their elution order on a polar column (VF‐5 ms).

^b^ Retention Time.

^c^ Linear Retention Indices measured on a polar column.

^d^ Linear Retention indices from literature; Data are means ± standard deviation of three (n = 3) replicates.

Three of these are unsaturated FAs, whereas six are saturated FAs. The total percentage of saturated FAs was almost double that of the unsaturated FAs. Palmitic acid was found to be the most abundant FA (54.1%), followed by oleic acid (21.1%) and elaidic acid (12.6%). The others were detected with an average percentage value ranging from 1.0% to 4.8%.

To the best of our knowledge, this is the first study on the FA content of this subspecies. Indeed, regarding this argument, there is no previous study on *T. chamaedrys*, whereas there are only a couple on other *Teucrium* species, but on the seed oils. In particular, Hachicha et al., [15] analyzed the seed oils of *Teucrium alopecurus* de Noé, *Teucrium nablii* S.Puech and *Teucrium polium* L. collected in Tunisia and extracted with *n*‐hexane reporting linoleic, linolenic, and palmitic acids as the major FAs whereas Smith et al. [16] analyzed the seed oil of *Teucrium depressum* Small extracted in Soxhlet with petroleum ether identifying three unusual trienoid components such as, all‐cis‐5, 9, 12‐octadecatrienoic acid (6.7%), trans‐5, ciss‐9, cis‐12‐octadecatrienoic acid (2.0%) and cis‐5, cis‐9‐octadecadienoic acid detected in trace amounts.

### 2.2 Volatile Composition

**FIGURE 2:**
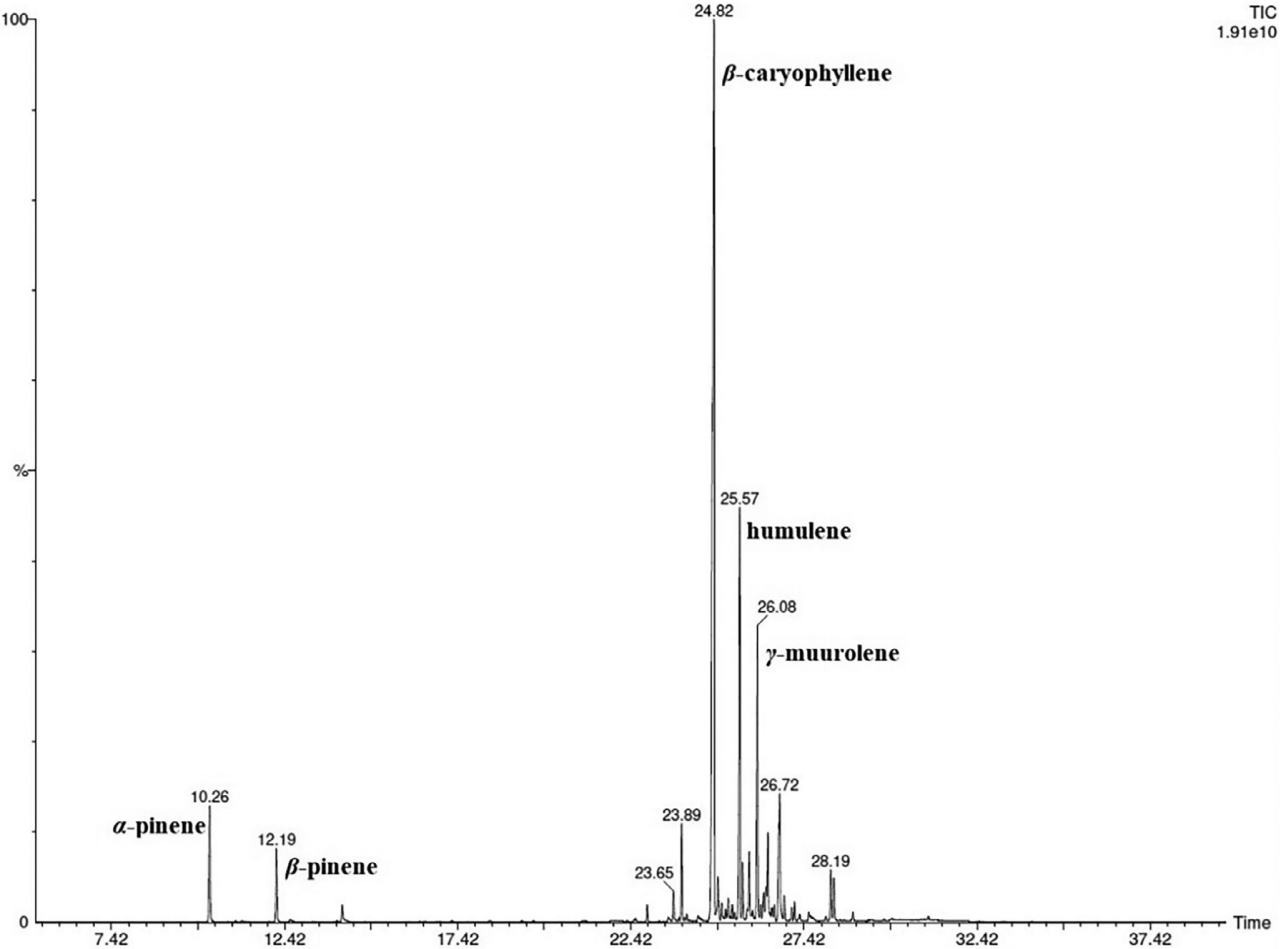
Gas chromatography‐mass spectrometry (GC‐MS) chromatogram of *T. chamaedrys* dried aerial parts extracted by headspace‐solid‐phase microextraction (HS‐SPME).

Headspace‐solid‐phase microextraction‐gas chromatography‐mass spectrometry (HS‐SPME‐GC‐MS) analysis allowed the identification of 26 volatile components from the dried aerial parts of *T. chamaedrys* subsp. *gracile* collected in Algeria (Table 2). The chromatogram is reported in Figure 2.

**TABLE 2:** Volatile content (percentage mean value ± standard deviation) of the dried aerial parts of *Teucrium chamaedrys* subsp. *gracile* as determined by headspace‐solid‐phase microextraction‐gas chromatography‐mass spectrometry (HS‐SPME‐GC‐MS).

N°	Component ^1^	RT ^a^	LRI ^b^	LRI ^c^	(%)
1	*α*‐pinene	10.26	938	942	4.5 ± 0.09
2	*α*‐fenchene	11.01	950	946	tr
3	Dehydrosabinene	11.09	958	955	tr
5	*β*‐pinene	12.19	967	970	2.4 ± 0.06
6	*β*‐myrcene	12.59	989	992	0.1 ± 0.01
7	Limonene	14.09	1041	1039	0.8 ± 0.03
8	*α*‐campholenal	17.26	1133	1132	tr
9	Pinocarvone	18.35	1150	1145	0.1± 0.01
10	Myrtenal	19.28	1168	1170	tr
11	Levoverbenone	19.61	1189	1191	tr
12	Carvacrol	21.98	1281	1278	0.3 ± 0.02
13	y‐langene	23.50	1377	1376	0.2 ± 0.02
14	(‐)‐*β*‐bourbonene	23.65	1391	1388	2.8 ± 0.02
15	*α*‐copaene	23.89	1405	1402	0.9 ± 0.04
16	*β*‐caryophyllene	24.82	1429	1424	51.5 ± 7.02
17	Humulene	25.57	1476	1473	14.9 ± 1.02
18	*γ*‐muurolene	26.08	1490	1486	9.7 ± 0.15
19	*β*‐cadinene	26.72	1510	1507	6.5± 0.08
20	cis‐calamenene	26.85	1533	1528	0.8 ± 0.03
21	*α*‐cadinene	27.15	1548	1544	0.6 ± 0.03
22	*α*‐calacorene	27.30	1550	1546	0.3 ± 0.02
23	Caryophyllene oxide	28.19	1585	1580	2.8 ± 0.07
24	*δ*‐cadinol	29.38	1623	1620	tr
25	Humulenol II	29.56	1635	1632	0.4 ± 0.02
26	Azulol	29.96	1779	1772	0.1 ± 0.01
	SUM				100.0
	Monoterpenes				8.2
	Sesquiterpenes				91.5
	Others				tr

^1^ The components are reported according to their elution order on a polar column (VF‐5 ms).

^a^ Retention Time.

^b^ Linear Retention Indices measured on a polar column.

^c^ Linear Retention indices from literature.

Data are means ± standard deviation of three (*n* = 3) replicates; tr: percentage mean values ˂0.1%.

The sesquiterpene fraction was significantly higher than the monoterpene fraction. β‐caryophyllene was the most abundant sesquiterpene, followed by humulene, γ‐muurolene, β‐cadinene, and caryophyllene oxide. Among monoterpenes, α‐pinene and β‐pinene were the most relevant, even if with average percentage values below 5%.

To the best of our knowledge, this is the first work reporting the characterization of the volatile fraction of this sub‐subspecies collected in Algeria. Previous studies have been conducted on the chemical composition of *T. chamaedrys* grown in other locations. For example, Giuliani et al. [17] determined the volatiles spontaneously emitted by leaves and flowers of this species cultivated at the Ghirardi Botanic Garden located in Northern Italy. Their results are partially in accordance with ours highlighting the sesquiterpenes β‐caryophyllene (27.1% in the leaves; 38.5% in the flowers) γ‐muurolene (22.8% in the leaves; 12.0% in the flowers) and β‐cubebene (12.3% in the leaves; 111.2% in the flowers), as the most abundant. In another study, Özel et al. [18] investigated the volatiles from the dried leaves of the nominal species collected in Turkey using the DTD–GC × GC–TOF/MS technique and the results showed that germacrene D (9.55%) was the most abundant sesquiterpene while α‐ and β‐pinene were the major monoterpenes. These results demonstrate that the chemical composition of the volatile fraction can vary depending on the growing location.

### 2.3 Non‐Volatile Composition

**FIGURE 3:**
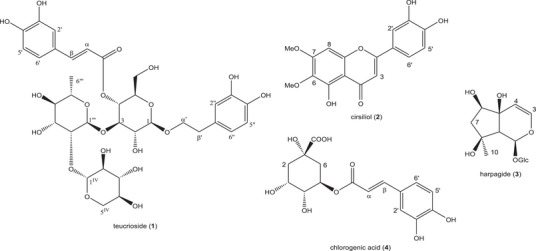
Structures of the non‐volatile compounds in *T. chamaedrys* subsp. *gracile* dried aerial parts.

The phytochemical analysis of the dried aerial parts of *T. chamaedrys* subsp. *gracile* collected in Algeria led to the identification of five non‐volatile compounds, namely teucrioside (**1**), cirsiliol (**2**), harpagide (**3**), chlorogenic acid (**4**), and quinic acid (**5**) (Figure 3).

These compounds belong to four different classes of natural compounds, such as phenyl‐ethanoid glycosides (**1**), flavonoids (**2**), iridoids (**3**), caffeoyl‐quinic acids (**4**).

To the best of our knowledge, these compounds were reported in this sub‐species for the first time during this study. In fact, teucrioside (**1**) has been previously identified only in the nominal species *T. chamaedrys* [12, 19] and in three of its subspecies, namely *Teucrium chamaedrys* subsp. *nuchense* (K.Koch) Rech.f. [20], *Teucrium chamaedrys* L. subsp. *chamaedrys* [21], and *Teucrium chamaedrys* subsp. *syspirense* (K.Koch) Rech.f. [22] plus in *Teucrium cavernarum* P.H.Davis [23]. This limited occurrence makes the compound an important chemophenetic marker at the sub‐species and species levels and eventually provides a discriminant with respect to the other species of the genus, even if further phytochemical analyses are necessary for this. Cirsiliol (**2**) has been previously isolated only from the nominal species [11] as well as in *T. capitatum* L. [4], *T. cavernarum* [23], *T. orientale* L. [24], and many other species of the genus [25]. This compound has also been identified in different genera of the family [26] and in other families [27, 28]. Given this wide occurrence, it has no chemophenetic value. It has been reported that this flavonoid has anti‐tumor effects in various tumors and anti‐inflammatory activity as it attenuates IL‐6‐induced cellular signaling by regulating Jak2 phosphorylation [29, 30]. The presence of harpagide (**3**) has been previously reported only from the nominal species [11] again, but also from different species of the genus [25, 31] and different genera of the family [31]. By the way, this compound is generally considered one of the main chemophenetic markers of the Lamiaceae family, and its occurrence in the studied species further confirms this. Yet, this compound has also been found in a few other families like Pedaliaceae and Scrophulariaceae [31]. In addition, harpagide has been observed to exert a wide number of biological activities such as cytotoxic, anti‐inflammatory, and neuroprotective [31]. Lastly, chlorogenic acid (**4**) has also been evidenced only in the nominal species [14] as well as in many other species of the genus [1]. It has also been found in different genera of the family [32, 33] as well as in different families [34, 35], and for this, it has no chemophenetic value. Further, chlorogenic acid has anti‐inflammatory, antibacterial, antioxidant, and antitumor effects [36]. The occurrence of all these non‐volatile compounds in *T. chamaedrys* subsp. *gracile* confirms its close chemophenetic relationship with the nominal species, but further phytochemical studies on its different populations are necessary to provide a wider understanding of its non‐volatile metabolite content.

## 3 Conclusions

This first phytochemical study on *Teucrium chamaedrys* subsp. *gracile* highlighted the presence of many metabolites belonging to different chemical classes. The results showed the presence of iridoid glucosides, a volatile profile rich in sesquiterpene compounds with β‐caryophyllene as the main component, and a FA content characterized mainly by the saturated fraction, where palmitic acid was the main constituent. These compositional data provide important information on the content of secondary metabolites in *T. chamaedrys*, highlighting the potential of this matrix as a natural source of bioactive molecules, thus adding a more complete chemophenic evaluation of the species and genus.

## 4 Experimental

### 4.1 Chemicals

The following chemicals and materials were utilized for this phytochemical study: 96 % ethanol for the extraction procedure by maceration; dichloromethane and high‐performance liquid chromatography (HPLC)‐grade methanol were used for lipid extraction; *n*‐butanol, distilled water and methanol as pure solvents or in mixture among them all to be used as eluting systems for the separation procedure of the total extract on open column chromatography using silica gel (40–63 µm) as stationary phase; 2N sulfuric acid for the developments of the thin‐layer chromatographies (TLCs); deuterated solvents (CD_3_OD and D_2_O) for the identification of the metabolites by means of nuclear magnetic resonance (NMR) spectroscopy; HPLC methanol for the identification of the metabolites by means of MS.

All the solvents having RPE purity grade, if not differently specified, together with the deuterated solvents, the TLCs, and HPLC‐grade methanol, were purchased from Merck (St. Louis, Missouri, USA), whereas silica gel was purchased from Fluka Analytical (Bergamo, Italy).

### 4.2 Plant Material

The aerial parts of *T. chamaedrys* subsp. *gracile* were collected in May 2022 at the Megriss region (Setif), situated in the north‐east of Algeria (36°19'44''N and 05°21'08''E at 1705 m altitude). The plant's harvesting station is a dry, slightly calcareous, high‐altitude lawn type. The plant material was dried in the shade in a ventilated room and identified by Professor Khellaf Rebbas from the Natural and Life Sciences Department, University of M'Sila, Algeria, by comparing the morphological features reported in literature [1]. A voucher specimen (Number N° KR0044) was deposited in the personal herbarium of Professor Khellaf Rebbas.

### 4.3 NMR and MS Instrumentation

The following instruments were used during this study: Rotavapor RII from Büchi (Cornaredo, Italy) for the evaporation procedure; UV lamp from Sigma Aldrich (Milan, Italy) at the wavelength of 254 nm for the visualization of TLCs.

NMR spectra were recorded at 298 K on a Bruker Avance II 400 MHz instrument (Billerica, Massachusetts, USA) The CD_2_HOD signal (p, 3.31 ppm) was set as reference for the spectra in CD_3_OD, whereas the HDO signal (s, 4.79 ppm) was set as reference for spectra in D_2_O.

MS spectra were acquired on a triple quadrupole mass spectrometer PE‐Sciex API‐3000 (Perkin Elmer Sciex, Toronto, ON, Canada), equipped with an ESI source operating in the negative and/or positive ion mode, in a mass spectral range of 100–1000 *m/z*. The capillary ion voltage was set at 5000 V for the positive ionization and ‐4500 V for the negative ionization. High‐purity nitrogen was used as curtain gas (5 L/min) and air as nebulizer (2 L/min) and drying gas (30 psi), with the temperature to heat the drying gas set at 100 °C. The flow rate of sample infusion was 20 µL/min. 20 acquisitions per sample were performed with the full width at half maximum (FWHM) set at *m/z* 0.7 ± 0.1 in each mass‐resolving quadrupole to operate with a unit resolution. Data were acquired and elaborated by Analyst 1.6 software (AB Sciex, Washington, USA). NMR spectra were reported in Figures S1‐S3.

### 4.4 Extraction Process and GC‐MS Determination of FAs Composition

For lipid extraction, maceration was performed according to the procedure described previously [37].

Briefly, the dried leaves (1 g) were added with 5 mL of dichloromethane/methanol (2:1, v/v) and macerated statically for 48 h at room temperature. The mixture was filtered with Whatman qualitative filter paper (Grade 3), and the extraction solvent was evaporated under reduced pressure at 30°C. The FA content of the sample was determined by the GC‐MS technique after transmethylation reaction using boron trifluoride in methanol. The analyses were performed by using a gas chromatograph coupled with a mass spectrometer, Clarus 500 model Perkin Elmer (Waltham, MA, USA), equipped with a flame ionization detector (FID). 2 µL of the extract was injected into the column (Varian Factor Four VF‐5) in splitless mode. The applied conditions of GC were the following: the injector was set to 280°C, and the oven temperature was set from 170°C with a rate of 3°C/min to 260°C for 15 min. The mass spectrometer was operated at 70 eV (EI) in full scan mode in the range 40–550 *m/z*. The ion source and the connection parts temperature were set at 180 and 200°C, respectively.

The identification of the FAs was performed by matching their mass spectra with those stored in the Wiley 2.2 and NIST 11 mass spectra libraries database and by calculating the linear retention indices (LRIs) using a series of alkane standards analyzed under the same conditions as those of the samples. LRIs were then compared with available retention data reported in the literature. The peak areas of the FID signal were used to calculate the relative concentrations of the components expressed as a percentage without the use of an internal standard and any factor correction. All analyses were carried out in triplicate.

### 4.5 HS‐SPME‐GC‐MS Analysis

The volatile profile of the dried aerial parts of *T. chamaedrys* subsp. *gracile* was characterized by using the HS‐SPME sampling technique. About 1 g of sample was placed inside a 20 mL glass vial with a polytetrafluoroethylene‐coated silicone septum.

A SPME device from Supelco (Bellefonte, PA, USA) with 1 cm fiber coated with 50/30 µm divinylbenzene/carboxen/polydimethylsiloxane was chosen to extract the components [38, 39].

Before use, the fiber was conditioned at 270°C for 30 min. The equilibration time for all samples was obtained by heating to 40°C for 10 min. After this time, the fiber was exposed to the headspace of the samples for 15 min at 40°C to capture and concentrate the volatiles. Lastly, the SPME fiber was inserted into the GC injector, maintained at 250°C in splitless mode for desorption of the collected components. The chromatographic analyses were carried out using the same apparatus described above. The capillary column was a Varian Factor Four VF‐5 (60m × 0.25 mm × 0.25). The oven conditions were set as follows: initially at 40°C, held for 10 min, then increased to 200°C at 2°C/min, then increased to 250°C at 2°C/min and held for 10 min. Helium was used as carrier gas at a constant rate of 1 mL/min.

The identification and quantification of the volatiles were performed as described in the previous section 4.4.

### 4.6 Extraction, Separation, and Identification of Non‐Volatile Metabolites

The extraction, separation, and identification of non‐volatile metabolites were conducted following a well‐established protocol in our laboratory [40].

A part of the collected aerial parts (2.0 g) was extracted with 500 mL of 96% ethanol till complete covering of the plant material. The whole was left to macerate for 14 days to have an exhaustive extraction of the plant material. At this point, the resulting solution was filtered, and the extracting solvent was removed at reduced pressure at a temperature of 50°C. During this step, the pH of the solution was checked to verify that it was not too acidic (<5.5) or too basic (>8.5), since an extreme acidity or alkalinity might cause unwanted secondary reactions like the hydrolysis of the ester and glycosidic bonds. In this case, pH was about 8. The obtained dried crude extract, coloured in light green, weighed 480 mg.

This whole extract was subjected to a separation procedure on column chromatography using 13 g of silica gel (ratio ∼ 1:35 w/w). The initial eluting system consisted of a mixture of *n*‐butanol and distilled water at the concentration ratio of 82:18 v/v (200 mL) but during the chromatographic run, this mixture was changed in order to increase its polarity and let the elution of the most polar compounds passing to a mixture of *n*‐butanol, methanol and distilled water in concentration ratio of 70:10:30 v/v/v (150 mL).

During this procedure, all the compounds were identified by comparison of their spectroscopic and spectrometric data with those reported in the literature: teucrioside (**1**) [12] as almost pure compound from the assembly of fractions 8–12 for the total weight of 21.2 mg; cirsiliol (**2**) and harpagide (**3**) [11] in mixture with saccharides in concentration ratio of 1:1 *w/w* from the assembly of fractions 34‐39 for the total weight of 30.5 mg; harpagide (**3**) [11]; chlorogenic acid (**4**) [41] in mixture with saccharides (ratio not calculable) from the methanol column wash for the total weight of 15.7 mg.

### 4.7 NMR and MS Data of the Identified Non‐Volatile Metabolites

Teucrioside (**1**): ^1^H NMR (CD_3_OD, 400 MHz) δ: 7.60 (1H, d, *J* = 15.9 Hz, H‐β), 7.06 (1H, d, *J* = 1.9 Hz, H‐2'), 6.96 (1H, dd, *J* = 8.3/1.9 Hz, H‐6'), 6.78 (1H, d, *J* = 8.3 Hz, H‐5'), 6.70 (1H, d, *J* = 2.0 Hz, H‐2''), 6.68 (1H, d, *J* = 8.0 Hz, H‐5''), 6.57 (1H, d, *J* = 8.0/2.0 Hz, H‐6''), 6.28 (1H, d, *J* = 15.9 Hz, H‐α), 5.40 (1H, d, *J* = 1.4 Hz, H‐1'''), 4.90‐4.82 (1H, overlapped with HDO signal, H‐1^IV^), 4.38 (1H, d, *J* = 7.9 Hz, H‐1), 4.05‐4.02 (2H, m, H‐α'), 4.00‐3.65 (overlapped saccharide signals), 2.82‐2.78 (2H, m, H‐β'), 1.07 (3H, d, *J* = 6.2 Hz, H‐6'''). ESI‐MS: *m/z* 755.8 [M—H]^‐^.

Cirsiliol (**2**): ^1^H NMR (D_2_O, 400 MHz) δ: 7.50 (1H, d, *J* = 1.5 Hz, H‐2'), 7.44 (1H, dd, *J* = 8.6/1.5 Hz, H‐6'), 7.01(1H, d, *J* = 8.5 Hz, H‐5'), 6.79 (1H, br s, H‐8), 6.63 (1H, br s. H‐3), 3.98 (3H, s, 7‐OMe), 3.85 (3H, s, 6‐OMe). ESI‐MS: *m/z* 353.7 [M + Na]^+^; *m/z* 329.4 [M—H]^‐^.

Harpagide (**3**):^1^H NMR (D_2_O, 400 MHz) δ: 6.32 (1H, d, *J* = 6.4 Hz, H‐3), 5.68 (1H, s, H‐1), 5.12 (1H, br d, *J* = 6.5 Hz, H‐4), 4.72‐4.68 (1H, overlapped with HDO signal, H‐1'), 3.85‐3.37 (overlapped saccharide signals), 2.50 (1H, s, H‐9), 1.95‐1.90 (1H, m, H‐7a), 1.80‐1.76 (1H, m, H‐7b), 1.26 (3H, s, H‐10). ESI‐MS: *m/z* 387.6 [M + Na]^+^.

Chlorogenic acid (**4**): ^1^H NMR (D_2_O, 400 MHz) δ: 7.72 (1H, d, *J* = 15.6 Hz, H‐β), 7.22 (1H, br s, H‐2'), 7.16 (1H, br d, *J* = 8.1, H‐6'), 6.95 (1H, d, *J* = 8.3 Hz, H‐5'), 6.40 (1H, d, *J* = 15.6 Hz, H‐α), 5.35‐5.32 (1H, m, H‐5), 4.28‐4.24 (1H, m, H‐4), 3.85‐3.75 (1H, overlapped with saccharide signals, H‐3), 2.07‐2.00 (4H, m, H‐2 and H‐6). ESI‐MS: *m/z* 353.4 [M—H]^‐^.

## Author Contributions

**Claudio Frezza**: methodology, formal analysis, investigation, writing – original draft, and writing – review and editing; **Daniela De Vita**: formal analysis, investigation, writing – original draft, and writing – review and editing. **Chiara Del Bosco**: formal analysis, investigation, writing – original draft, and writing – review and editing. **Khellaf Rebbas**: resources. **Hamdi Bendif**: writing – original draft and writing – review and editing. **Stefania Garzoli**: conceptualization, investigation, formal analysis, writing original draft, writing – review and editing, and supervision. All authors have read and agreed to the published version of the manuscript.

## Conflicts of Interest

The authors declare no conflicts of interest.

## Supporting information




**Supporting File 1**: cbdv70259‐sup‐0001‐SuppMat.pdf

## Data Availability

All data are reported in the manuscript

## References

[1] M. Stanković , Teucrium Species: Biology and Applications (Springer, 2020).

[2] P. Quezel and S. Santa , Nouvelle flore de l'Algérie et des régions désertiques méridionales (du Centre Nat. de la Recherche Scientifique, 1963).

[3] C. Frezza , D. De Vita , O. Giampaoli , et al., “Phytochemical Analysis on the Leaves of *Teucrium capitatum* L. subsp. *capitatum* Collected in the Botanical Garden of Rome,” Annals of Botany 14 (2024): 97–102.

[4] C. Frezza , G. Bozzato , F. Sciubba , et al., “Phytochemical analysis on the aerial parts of *Teucrium capitatum* L. With aspects of chemosystematics and ethnobotany,” Natural Product Research 37, no. 14 (2023): 2398–2407. 35648096 10.1080/14786419.2022.2081967

[5] D. Larrey , T. Vial , A. Pauwels , et al., “Hepatitis After Germander (*Teucrium chamaedrys*) Administration: Another Instance of Herbal Medicine Hepatotoxicity,” Annals of Internal Medicine 117, no. 2 (1992): 129–132. 1605427 10.7326/0003-4819-117-2-129

[6] J. Loeper , V. Descatoire , P. Letteron , et al., “Hepatotoxicity of Germander in Mice,” Gastroenterology 106, no. 2 (1994): 464–472. 8299912 10.1016/0016-5085(94)90606-8

[7] Available from, https://powo.science.kew.org/taxon/urn:lsid:ipni.org:names:77188408‐1 Accessed: May 2025.

[8] A. Muselli , J.‐M. Desjobert , J. Paolini , et al., “Chemical Composition of the Essential Oils of *Teucrium chamaedrys* L. From Corsica and Sardinia,” Journal of Essential Oil Research 21, no. 2 (2009): 138–143.

[9] A. Ulubelen , A. Topcu , and Ü. Kaya , “Steroidal compounds From *Teucrium chamaedrys* subsp. *chamaedrys* ,” Phytochemistry 36, no. 1 (1994): 171–173.

[10] E. Bedir , R. Manyam , and I. A. Khan , “Neo‐clerodane Diterpenoids and Phenylethanoid Glycosides From *Teucrium chamaedrys* L.,” Phytochemistry 63, no. 8 (2003): 977–983. 12895549 10.1016/s0031-9422(03)00378-9

[11] C. Frezza , A. Venditti , G. Matrone , et al., “Iridoid Glycosides and Polyphenolic Compounds From *Teucrium chamaedrys* L.,” Natural Product Research 32, no. 13 (2018): 1583–1589. 29058476 10.1080/14786419.2017.1392948

[12] G. A. Gross , M. F. Lahloub , C. Anklin , H. R. Schulten , and O. Sticher , “Teucrioside, a Phenylpropanoid Glycoside From *Teucrium chamaedrys* ,” Phytochemistry 27, no. 5 (1988): 1459–1463.

[13] S. Pacifico , B. D'Abrosca , M. T. Pascarella , et al., “Antioxidant Efficacy of Iridoid and Phenylethanoid Glycosides From the Medicinal Plant *Teucrium chamaedris* in Cell‐Free Systems,” Bioorganic & Medicinal Chemistry 17, no. 17 (2009): 6173–6179. 19674906 10.1016/j.bmc.2009.07.065

[14] L. Vlase , D. Benedec , D. Hanganu , et al., “Evaluation of Antioxidant and Antimicrobial Activities and Phenolic Profile for Hyssopus officinalis, Ocimum basilicum and *Teucrium chamaedrys* ,” Molecules 19 (2014): 5490–5507. 24786688 10.3390/molecules19055490 PMC6270679

[15] S. F. Hachicha , S. Barrek , T. Skanji , H. Zarrouk , and Z. G. Ghrabi , “Fatty Acid, Tocopherol, and Sterol Content of three *Teucrium* species From Tunisia,” Chemistry of Natural Compounds 45 (2009): 304–308.

[16] C. R. Smith , R. M. Freidinger , J. W. Hagemann , G. F. Spencer , and I. A. Wolff , “ *Teucrium depressum* Seed Oil: A New Source of Fatty Acids With Δ‐unsaturation,” Lipids 4, no. 6 (1969): 462–465. 5367928 10.1007/BF02531025

[17] C. Giuliani , M. Bottoni , R. Ascrizzi , et al., “Morphology and Phytochemistry of *Teucrium chamaedrys* L. (Lamiaceae) Cultivated at the Ghirardi Botanic Garden (Lombardy, Northern Italy),” Flora 282 (2021): 151898.

[18] M. Z. Özel , F. Göğüş , and A. C. Lewis , “Determination of *Teucrium chamaedrys* Volatiles by Using Direct Thermal Desorption–Comprehensive Two‐dimensional Gas Chromatography–Time‐of‐Flight Mass Spectrometry,” Journal of Chromatography A 1114, no. 1 (2006): 164–169. 16516906 10.1016/j.chroma.2006.02.036

[19] L.‐Z. Lin , J. M. Harnly , and R. Upton , “Comparison of the Phenolic Component Profiles of Skullcap (*Scutellaria lateriflora*) and Germander (*Teucrium canadense* and *T. chamaedrys*), a Potentially Hepatotoxic Adulterant,” Phytochemical Analysis 20 (2009): 298–306. 19402188 10.1002/pca.1127 PMC3583524

[20] T. Korkotadze , D. Berashvili , S. Gokadze , et al., “Phytochemical Characterization of the Aerial Parts of *Teucrium nuchense* K. Koch: An Endemic Species of Caucasian Flora,” Natural Products Journal 15, no. 7 (2025): e22103155317820, 10.2174/0122103155317820240827071846.

[21] S. Piccolella , M. Scognamiglio , B. D'Abrosca , A. Esposito , A. Fiorentino , and S. Pacifico , “Chemical Fractionation Joint to In‐Mixture NMR Analysis for Avoiding the Hepatotoxicity of *Teucrium chamaedrys* L. subsp. chamaedrys,” Biomolecules 11, no. 5 (2021): 690. 34063021 10.3390/biom11050690 PMC8148020

[22] I. Çalis , E. Bedir , A. D. Wright , and O. Sticher , “Neoclerodane Diterpenoids From *Teucrium chamaedrys* subsp. syspirense,” Journal of Natural Products 59 (1996): 457–460.

[23] F. Göger , A. Kaya , M. Dinç , and S. Doğu , “Phenolic Compounds Determination and Antioxidant Activity of *Teucrium cavernarum* ,” Eskişehir Technical University Journal of Science and Technology C‐ Life Sciences and Biotechnology 8, no. 2 (2019): 229–237.

[24] G. Oganesyan , “Flavonoids From Teucrium orientale,” Chemistry of Natural Compounds 49, no. 1 (2013): 106–107.

[25] J. B. Harborne , F. A. Tomás‐Barberán , C. A. Williams , and M. I. Gil , “A Chemotaxonomic Study of Flavonoids From European *Teucrium* Species,” Phytochemistry 25, no. 12 (1986): 2811–2816.

[26] F. A. Tomás‐Barberán and E. Wollenweber , “Flavonoid Aglycones From the Leaf Surfaces of Some Labiatae Species,” Plant Systematics and Evolution 173 (1990): 109–118.

[27] E. Wollenweber , “Flavones and Flavonols,” in The Flavonoids—Advances in Research Since 1986, ed. J. B. Harborne (Chapman and Hall, 1994): 259–335.

[28] D. K. Patel , “Biological Importance, Therapeutic Benefit, and Medicinal Importance of Flavonoid, Cirsiliol for the Development of Remedies Against Human Disorders,” Current Bioactive Compounds 18, no. 3 (2022): 2–10.

[29] M. Luo , Z. Su , H. Gao , et al., “Cirsiliol Induces Autophagy and Mitochondrial Apoptosis Through the AKT/FOXO1 Axis and Influences Methotrexate Resistance in Osteosarcoma,” Journal of Translational Medicine 21, no. 1 (2023): 907, 10.1186/s12967-023-04682-7. 38087310 PMC10714637

[30] H. J. Lim , H. J. Jang , S. G. Bak , et al., “In Vitro Inhibitory Effects of Cirsiliol on IL‐6‐induced STAT3 Activation Through Anti‐inflammatory Activity,” Bioorganic & Medicinal Chemistry Letters 29, no. 13 (2019): 1586–1592, 10.1016/j.bmcl.2019.04.053. 31060888

[31] C. Frezza , D. de Vita , C. Toniolo , et al., “Harpagide: Occurrence in Plants and Biological Activities—A Review,” Fitoterapia 147 (2020): 104764. 33122133 10.1016/j.fitote.2020.104764

[32] G. Zgórka and K. Głowniak , “Variation of Free Phenolic Acids in Medicinal Plants Belonging to the Lamiaceae family,” Journal of Pharmaceutical and Biomedical Analysis 26 (2001): 79–87. 11451645 10.1016/s0731-7085(01)00354-5

[33] A. Moshari‐Nasirkandi , A. Alirezalu , H. Alipour , and J. Amato , “Screening of 20 Species From Lamiaceae Family Based on Phytochemical Analysis, Antioxidant Activity and HPLC Profiling,” Scientific Reports 13 (2023): 16987. 37813985 10.1038/s41598-023-44337-7 PMC10562417

[34] V. V. Milevskaya , S. Prasad , and Z. A. Temerdashev , “Extraction and Chromatographic Determination of Phenolic Compounds From Medicinal Herbs in the Lamiaceae and Hypericaceae Families: A Review,” Microchemical Journal 145 (2019): 1036–1049.

[35] V. Marques and A. Farah , “Chlorogenic Acids and Related Compounds in Medicinal Plants and Infusions,” Food Chemistry 113 (2009): 1370–1376.

[36] L. Wang , X. Pan , L. Jiang , et al., “The Biological Activity Mechanism of Chlorogenic Acid and Its Applications in Food Industry: A Review,” Frontiers in Nutrition 9 (2022): 943911, 10.3389/fnut.2022.943911. 35845802 PMC9278960

[37] M. T. Frangipane , S. Garzoli , D. De Vita , D. R. Massantini , and P. Corona , “Identifying the Sensory Profile and Fatty Acid Composition for Quality Valorization of Marrone Chestnut Cultivars,” European Food Research and Technology 250 (2024): 2837–2847.

[38] C. Taiti , G. Di Matteo , M. Spano , et al., “Metabolomic Approach Based on Analytical Techniques for the Detection of Secondary Metabolites From *Humulus lupulus* L. Dried Leaves,” International Journal of Molecular Sciences 24 (2023): 13732. 37762036 10.3390/ijms241813732 PMC10531422

[39] S. Vitalini , M. Di Martile , V. Cicaloni , et al., “Volatile and Non‐Volatile Content Determination and Biological Activity Evaluation of Fresh *Humulus lupulus* L. (cv. Chinook) Leaves and Inflorescences,” Separations 10 (2023): 91.

[40] C. Frezza , D. De Vita , A. Venditti , et al., “Phytochemical Analysis and Biological Activities of the Aerial Parts of *Odontites vulgaris* Moench,” Fitoterapia 175 (2024): 105936. 38552807 10.1016/j.fitote.2024.105936

[41] I. Berregi , J. I. Santos , G. D. Campo , J. I. Miranda , and J. M. Aizpurua , “Quantitation Determination of Chlorogenic Acid in Cider Apple Juices by ^1^H NMR Spectrometry,” Analytica Chimica Acta 486, no. 2 (2003): 269–274.

