# Supplementary material for: Unveiling the Chemical Profile of Teucrium chamaedrys subsp. gracile (Batt.) Rech.F. dried Aerial Parts From Algeria
Source: Chem Biodivers. 2025 Jul 17;22(11):e00236. doi: 10.1002/cbdv.202500236 (PMC12629161; doi:10.1002/cbdv.202500236)
Supplement: Supplementary file 1 — Supporting File 1: cbdv70259‐sup‐0001‐SuppMat.pdf [file CBDV-22-e00236-s001.pdf]

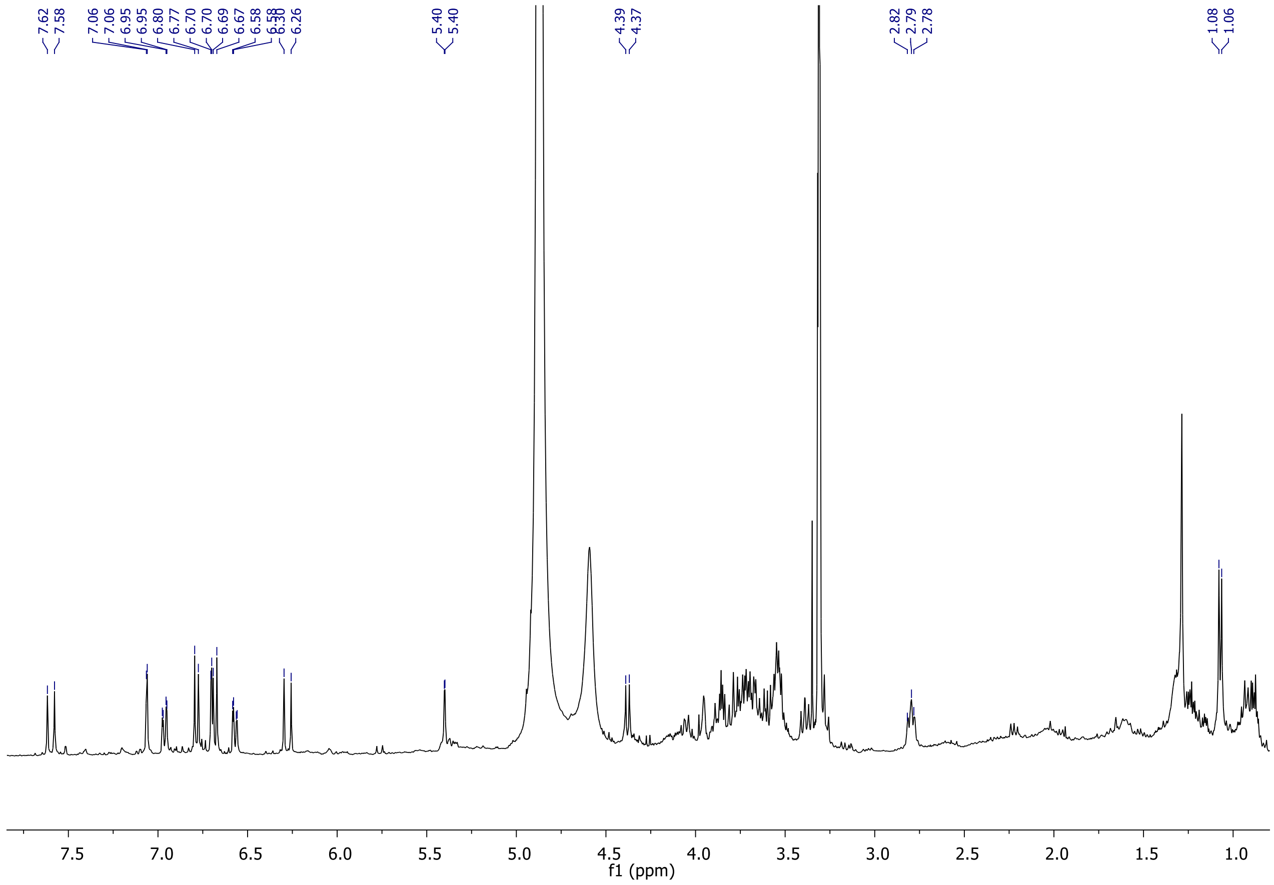

Figure S1: ^{1}H NMR of the assembly of fractions 8-12 from column 2

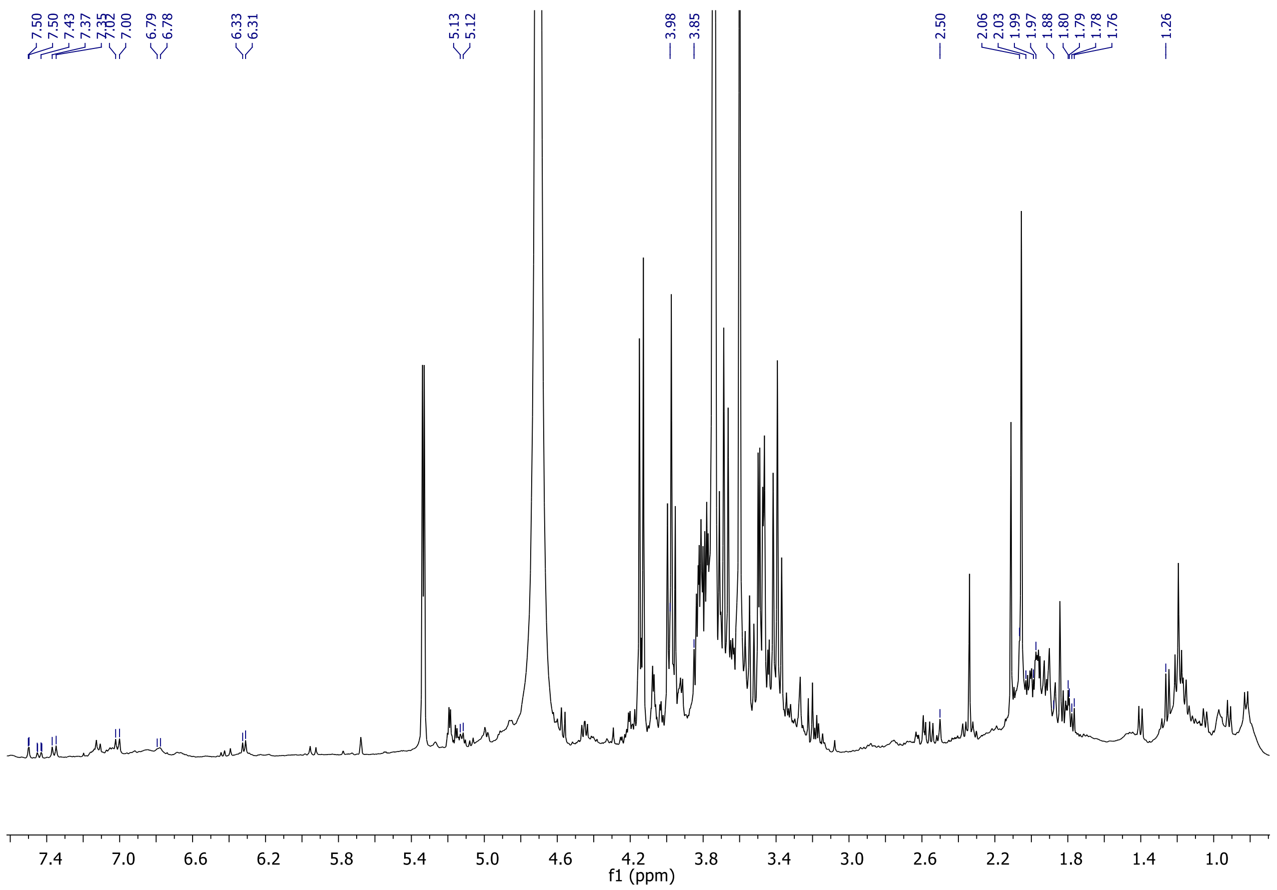

Figure S2: ^{1}H NMR of the assembly of fractions 34-39 from column 2

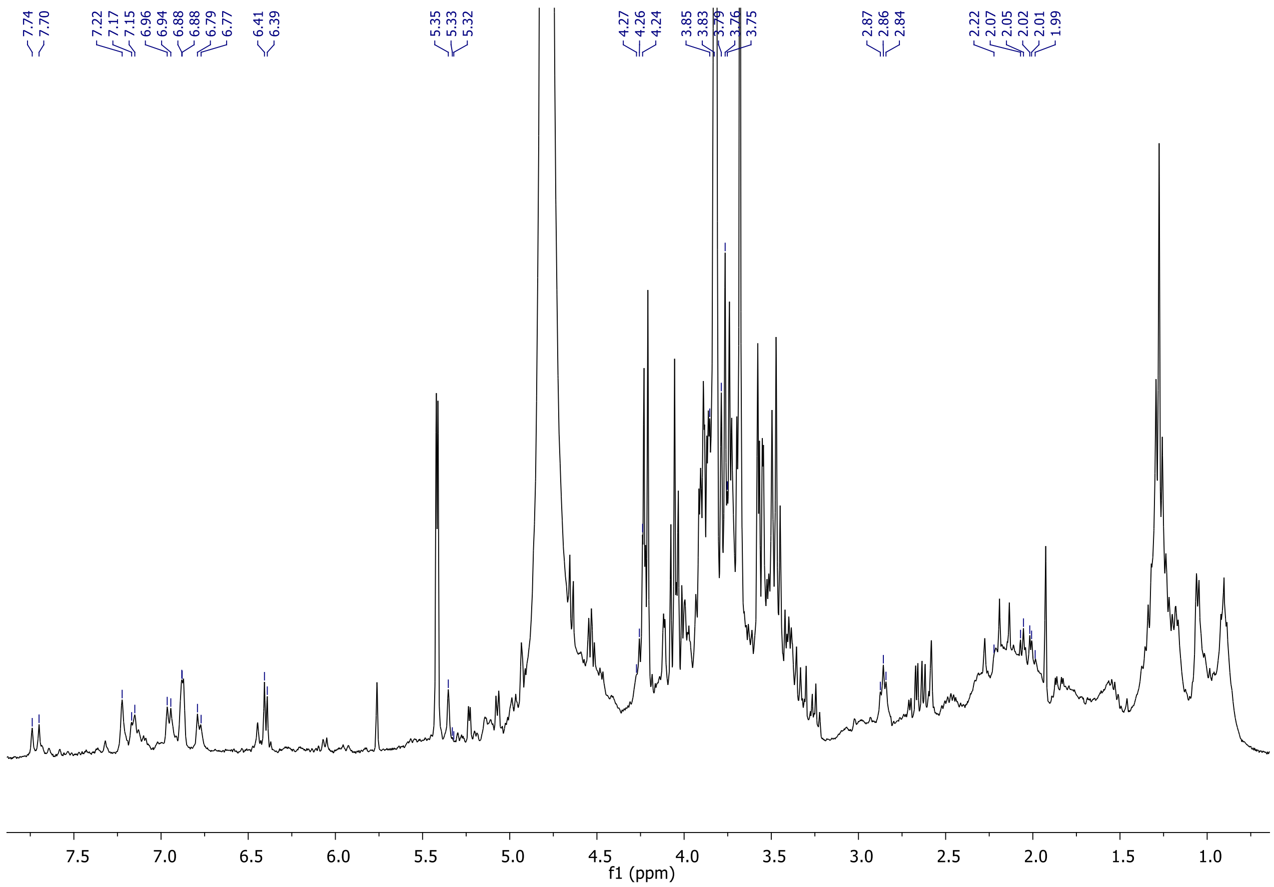

Figure S3: ^{1}H NMR of the column 2 methanol wash
